# Supplementary figures and images for: Intrusive social support among Black and White individuals with type 2 diabetes: A “Control issue” or a sign of “Concern and love”?
Source: PLoS One. 2023 Aug 8;18(8):e0288258. doi: 10.1371/journal.pone.0288258 (PMC10409292; doi:10.1371/journal.pone.0288258)

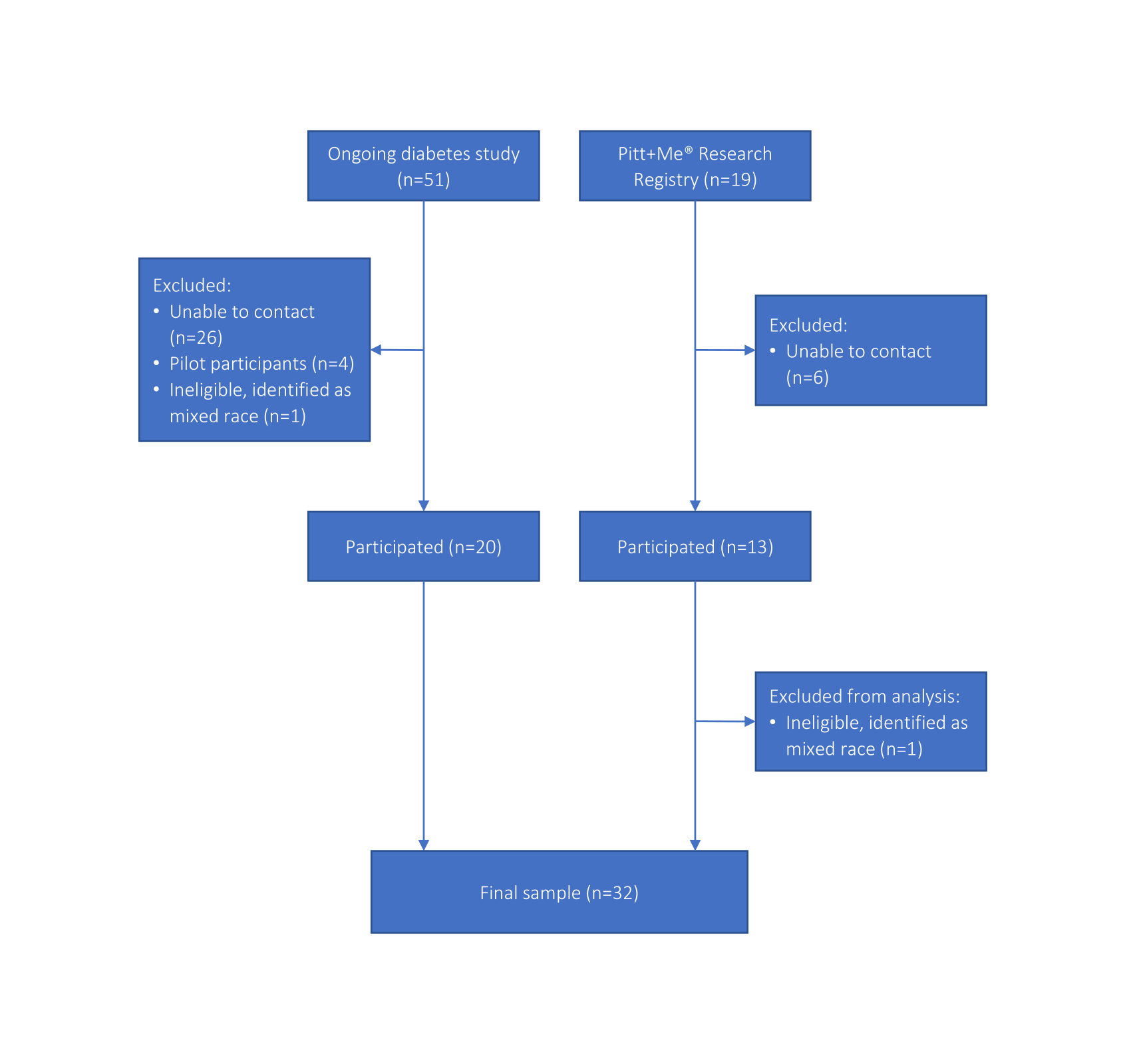

Supplement: S1 Fig — (TIF) [file pone.0288258.s001.tif]
