# Supplementary material for: Intrusive social support among Black and White individuals with type 2 diabetes: A “Control issue” or a sign of “Concern and love”?
Source: PLoS One. 2023 Aug 8;18(8):e0288258. doi: 10.1371/journal.pone.0288258 (PMC10409292; doi:10.1371/journal.pone.0288258)
Supplement: S1 File — (PDF) [file pone.0288258.s002.pdf]

S1 File. Interview Questions.

Warm-up Questions

Let's start with a few questions just for me to get to know you a little bit better.

- How are you doing today? How are things going with COVID? Are you currently working?
- When were you first diagnosed with diabetes?
  - How did you feel? What were your initial thoughts?
- In regards to diabetes, how have things been going lately?

Main Questions

1. What are you currently doing to take care of diabetes?
   Probe for other activities, e.g. checking blood sugar, taking medication, going to doctor's appointments

Now, we're going to shift into another part of the interview where I'm going to ask you questions about interactions with your family members regarding diabetes. The questions I'm going to ask might feel a little bit repetitive, but this is because we really want to understand all the details (big and small) about how you interact with your family regarding diabetes. So, feel free to be as detailed as you would like to be. First, in order to figure out which family members we're going to talk about, I'm going to show you a picture with three circles.

Display picture via Zoom screen sharing OR have them open the image in their email and make sure they can see it.

First, I want you to think of all your family members, including people who may not be blood related but that you consider to be family. Think of them as this set of circles and imagine yourself at the center of these circles where it says "YOU."

The inner circle represents people who you consider to be family that you feel so close to that it's hard to imagine life without them. The middle circle is the next closest group of people that are like family to you, and the outer circle are the group of people who are least close to you but are still part of your family network.

For the rest of the interview, we're going to focus on people that are in the inner circle. Again, these are people who are like family to you that you feel so close to that it's hard to imagine life without them. Could you tell me the first names and your relationships to the people that would go in this inner circle?

Okay, so you said that *[list names and relationships]* are in your inner circle. Anyone else? (If uncomfortable with saying their names aloud, you can ask them the next question.)
Probe to ask for children's ages

Alright, great. So now, I'm going to ask you questions about your interactions with the people in your inner circle in relation to diabetes, and you can think of the people you mentioned while answering these questions. *(Stop sharing screen)*
Probe to make sure they discuss everyone in their inner circle when answering questions
Probe for more specific examples – we want to know who initiated the support, how they felt about it, why certain things happened, all the minute details

1. How have the people in your inner circle responded to you having diabetes?

2. What do your conversations about diabetes with the people in your inner circle usually look like? Who initiates these conversations?

3. Of the people in your inner circle, who do you feel supports you regarding diabetes?
    a. What does that help look like? How do you feel about that?

4. Are there (other) ways that the people in your inner circle try to help you take care of diabetes? If so, what do they do?
   Probe to make sure they discuss both what they <u>say</u> and <u>do</u> to help

5. Are there (other) ways that the people in your inner circle try to support you emotionally about having diabetes? If so, what do they say or do?
   Probe to make sure they discuss both what they <u>say</u> and <u>do</u> to help
   Do they ever encourage you about taking care of diabetes? Do they ever reassure you about how things are going with diabetes?

6. So far, you've mentioned that they (restate some behaviors they mentioned). Is there anything that your inner circle says or does that makes it more difficult for you to take care of diabetes? If so, what do they do?
   Probe to make sure they discuss both what they say and do to help

7. Is there anything that your inner circle says or does that makes it easier for you to take care of diabetes? If so, what do they do?
   Probe to make sure they discuss both what they say and do to help

Thank you so much for everything you've shared so far. Now, I want to ask you about some specific concepts that researchers studying this topic have developed.

8. In previous studies, researchers have observed different kinds of support that close others might give to people with diabetes. Sometimes, the people close to you might try to support you with diabetes *without you asking them to*. For example, someone might suggest that you eat particular foods, or compliment you on how you're handling diabetes, without you asking them to do that.
   a. Have the people in your inner circle ever tried to support you without you asking them to? If so, can you tell me about the most recent time that this happened? How did you feel at that time?
   b. Are there ever times when this behavior ends up being a good thing/bad thing? What makes the difference between it being a good thing/bad thing?
   c. (If there is a discrepancy): I'd like to check in on something to make sure I understand things correctly the way you see them... Earlier you said (restate activity), which seemed to me like it might be an example of them giving you support without you asking them to, but I noticed you didn't bring it up here. Can you tell me, do you think is an example of this kind of behavior, or does it seem different to you?
   d. If this has never happened to you, how do you think you would feel if it did?

9. Now, I want to ask you about another kind of support that researchers have observed. With this kind of support, people close to you might try to do things for you that you could do yourself, or they might try to restrict your ability to do certain things. For example, someone might shop for food at the grocery store for you, or they might try to keep you from eating certain foods.

a. Have the people in your inner circle ever done things for you or tried to restrict you from doing certain things? If so, can you tell me about the most recent time that this happened? How did you feel at that time?
b. Are there ever times when this behavior ends up being a good thing/bad thing? What makes the difference between it being a good thing/bad thing?
c. (If there is a discrepancy): I'd like to check in on something to make sure I understand things correctly the way you see them... Earlier you said (restate activity), which seemed to me like it might be an example of them giving you support without you asking them to, but I noticed you didn't bring it up here. Can you tell me, do you think is an example of this kind of behavior, or does it seem different to you?
d. If this has never happened to you, how do you think you would feel if it did?

10. In what ways would you want to be supported by the people in your inner circle? How much do you want your inner circle to be involved in diabetes?

11. In what ways would you not want to be supported by the people in your inner circle?

12. Earlier on in the interview, I showed you that diagram of the social circles, and I’ve only been asking about your inner circle. I wanted to check in, though, about the middle and outer circles: Do the people in your other social circles support you with diabetes in ways that are different than those in your inner circle? Is there ever a lack of support from these middle and outer circles regarding diabetes?

So we’re nearing the end of the interview portion of the study – I just have a few more questions for you.

Wrap-up questions

- What are some ways that you think things have gotten better with diabetes?
- Is there anything else that you thought about while we were talking that you didn’t get a chance to talk about?

Probes

General:
- Can you say a little bit more?
- What do you mean by ______?
- Can you give me an example?
- Is this example typical for you?
- How do you feel about that?
- What sorts of conversations do you have about _____?
- Who initiated the conversation?
- What sparked the conversation?

About other people's behaviors:
- How do you know they feel _________?
- Do they do or say something that makes you think that?
- How helpful is it when they do ______?
- How do you feel when they do _______?
- Would you rather them do something else?

Why questions:
- Is there a particular reason why ________?

Summarizing:
- It sounds like that made you ______ (feeling).
- It sounds like ______ (specific example). Is that right?

Change:
- Has it always been this way?
- When did things start to change?
- Has the way your inner circle supported you changed over time?

Random:
- How do the people in your inner circle react to highs/lows?
- How does diabetes usually come up when talking to people in your inner circle?
- Can you tell me about a recent problem you had with diabetes where someone in your inner circle was involved?
- (If not receiving support from their inner circle): Does your inner circle support you in other domains of your life? How so?
