# Supplementary material for: Intrusive social support among Black and White individuals with type 2 diabetes: A “Control issue” or a sign of “Concern and love”?
Source: PLoS One. 2023 Aug 8;18(8):e0288258. doi: 10.1371/journal.pone.0288258 (PMC10409292; doi:10.1371/journal.pone.0288258)
Supplement: S2 File — (PDF) [file pone.0288258.s003.pdf]

Code System

Code System	Frequency
Code System	6528
Diagnosis circumstances	63
Inner circle feelings about diabetes	0
Unaffected	7
Acceptance	9
Avoidance	1
Sad	4
Frustrated	6
Upset	1
Guilt/Blame	3
Concerned	42
Content	6
Surprised	3
Not surprised	2
Other feeling	1
Current diabetes feelings	0
Overwhelmed	5
Unaffected	2
Acceptance	5
Avoidance	4

Sad	9
Frustrated	6
Upset	2
Concerned	14
Guilt/Blame	0
Content	29
Other feeling	2
Support receipt	0
Support received	518
No support received	246
Type of support	0
Instrumental	0
Buying groceries	59
Preparing meals	69
Offer to help	43
Other instrumental	52
Informational	0
Advice/Guidance	207
Other informational	8
Emotional	0
Encouragement	36
Expressing love/care	6
Listening	10

Validation	8
Other emotional	27
Expressing concern	7
Monitoring	119
Asking about well-being	59
Generic/Other type	265
Inner circle source of behavior	0
Spouse/partner	198
Parent	40
Sibling/step-sibling	106
Child/step-child	111
Grandchild/step-grandchild	8
Grandchildren/step-grandchildren	3
Other family member	66
General family/inner circle	333
Friend/co-worker	75
Support communication	0
Verbal	398
Nonverbal	181
Emotional tone of support	0
Concerned	45
Warm	58
Judging/criticizing	108

Neutral	21
Unclear	243
Other tone	1
Mode of support delivery	0
Suggesting	72
Inquiring	139
Telling	208
"Problematic" support	0
Restrictions	0
Implicit	14
Explicit	62
Doing things for them	0
Implicit	122
Explicit	52
Unsolicited support	0
Implicit	414
Explicit	103
Feelings about network member behavior	0
Desired	189
Undesired	161
Unaffected	28
Acceptance	31
Sad	2

Frustrated	24
Upset	8
Guilt/Blame	5
Ambivalent	66
Other feeling	4
Reasons for network member behavior	0
Expressing love/care/concern	86
Don't talk about diabetes	3
Not an emotional issue	7
Already taking care of diabetes	14
Don't ask for support	2
Network characteristics	0
Diabetes knowledge	31
Trait characteristics	24
State characteristics	17
Participant characteristics	0
Trait characteristics	19
State characteristics	9
Relationship characteristics	42
Other reason	18
Reasons for feelings about network member behavior	0
Expresses love/care/concern	36
Frequency of support	14

Mode of delivery/emotional tone	19
Need the help	21
Makes things easier	4
Not wanting to burden others	8
Already taking care of diabetes	3
Network characteristics	0
Knowledge about diabetes	6
Trait characteristics	7
State characteristics	10
Participant characteristics	0
Trait characteristics	44
State characteristics	20
Relationship characteristics	10
Other reason	14
Collaboration on diabetes activities	70
Network member behaviors not facilitate diabetes management	61
Outer circle source of behavior	0
Spouse/partner	0
Parent	0
Sibling/step-sibling	2
Child/step-child	3
Grandchildren/step-grandchildren	0
Other family member	6

General family/outer circle	23
Friend/co-worker	24
Changes	0
Diabetes feelings	7
Diabetes support behavior	25
Division of labor	24
Impacts of COVID	0
Diabetes management	13
Relationships/Communication	29
Misc	0
Asking about emotional support	38
Don't talk about diabetes	50
