# Supplementary material for: Intrusive social support among Black and White individuals with type 2 diabetes: A “Control issue” or a sign of “Concern and love”?
Source: PLoS One. 2023 Aug 8;18(8):e0288258. doi: 10.1371/journal.pone.0288258 (PMC10409292; doi:10.1371/journal.pone.0288258)
Supplement: Attachment — Submitted filename: Response to Reviewers.docx [file pone.0288258.s004.docx]

PONE-D-23-00304

***We thank the editor and the reviewers for their time and effort in providing us with feedback on the current manuscript. Our response to each comment is italicized and bolded.***

Journal requirements:

When submitting your revision, we need you to address these additional requirements.

1. Please ensure that your manuscript meets PLOS ONE's style requirements, including those for file naming. The PLOS ONE style templates can be found at

[https://journals.plos.org/plosone/s/file?id=wjVg/PLOSOne_formatting_sample_main_body.pdf](https://urldefense.com/v3/__https:/journals.plos.org/plosone/s/file?id=wjVg*PLOSOne_formatting_sample_main_body.pdf__;Lw!!LLK065n_VXAQ!m7EDcJO_qvNMzAW5J2VFwpom5QgS8ZuqJuGMBNrX_oAhJO6UDxSbBmlloCVTbQe-0nfowWBlsTOq6az4WRE$) and

[https://journals.plos.org/plosone/s/file?id=ba62/PLOSOne_formatting_sample_title_authors_affiliations.pdf](https://urldefense.com/v3/__https:/journals.plos.org/plosone/s/file?id=ba62*PLOSOne_formatting_sample_title_authors_affiliations.pdf__;Lw!!LLK065n_VXAQ!m7EDcJO_qvNMzAW5J2VFwpom5QgS8ZuqJuGMBNrX_oAhJO6UDxSbBmlloCVTbQe-0nfowWBlsTOqSeneyYw$)

***We have reviewed the manuscript and supporting information and ensured that they now meet PLOS ONE’s style requirements.***

2. Please provide additional details regarding participant consent. In the ethics statement in the Methods and online submission information, please ensure that you have specified what type you obtained (for instance, written or verbal, and if verbal, how it was documented and witnessed). If your study included minors, state whether you obtained consent from parents or guardians. If the need for consent was waived by the ethics committee, please include this information.

***We have now added this information to the Method section of the manuscript. Participants signed the consent form online, and we are unsure whether this qualifies as written or verbal.***

3. Thank you for stating the following in the Acknowledgments Section of your manuscript:

"This research was supported by National Institutes of Health F31 MD015922 and the Society for Personality and Social Psychology through the Jenessa Shapiro Graduate Research Award. The authors are indebted to Tiona Jones, Jennifer Melnyk, and Abigail Vaughn for assistance with recruitment; to Melissa Zajdel, Fiona Horner, Karen Lincoln, Aidan Wright, Kasey Creswell, Sheldon Cohen, Brooke Feeney, and Michael Trujillo for feedback on the study design; and to the participants who gave their time to this study."

We note that you have provided additional information within the Acknowledgements Section that is not currently declared in your Funding Statement. Please note that funding information should not appear in the Acknowledgments section or other areas of your manuscript. We will only publish funding information present in the Funding Statement section of the online submission form.

***Thank you for catching this – we have removed any mention of funding sources from the Acknowledgements and ensured that they are present in the Funding Statement.***

Please remove any funding-related text from the manuscript and let us know how you would like to update your Funding Statement. Currently, your Funding Statement reads as follows:

"JN received funding from the National Institute on Minority Health and Health Disparities ([https://www.nimhd.nih.gov/](https://urldefense.com/v3/__https:/www.nimhd.nih.gov/__;!!LLK065n_VXAQ!m7EDcJO_qvNMzAW5J2VFwpom5QgS8ZuqJuGMBNrX_oAhJO6UDxSbBmlloCVTbQe-0nfowWBlsTOqtBtcbo0$)) F31MD015922 and the Society for Personality and Social Psychology ([https://spsp.org/](https://urldefense.com/v3/__https:/spsp.org/__;!!LLK065n_VXAQ!m7EDcJO_qvNMzAW5J2VFwpom5QgS8ZuqJuGMBNrX_oAhJO6UDxSbBmlloCVTbQe-0nfowWBlsTOqxsozJjo$)) Jenessa Shapiro Graduate Research Award. The funders had no role in study design, data collection and analysis, decision to publish, or preparation of the manuscript."

***We have updated the Funding Statement to include an additional funding source – the National Heart, Lung, and Blood Institute (***[***https://www.nhlbi.nih.gov/***](https://www.nhlbi.nih.gov/)***) T32HL079891.***

Please include your amended statements within your cover letter; we will change the online submission form on your behalf.

3. Please include your full ethics statement in the ‘Methods’ section of your manuscript file. In your statement, please include the full name of the IRB or ethics committee who approved or waived your study, as well as whether or not you obtained informed written or verbal consent. If consent was waived for your study, please include this information in your statement as well.

***As mentioned above, we have now added this information to the Method section of the manuscript. Participants signed the consent form online, and we are unsure whether this qualifies as written or verbal.***

4. We noted in your submission details that a portion of your manuscript may have been presented or published elsewhere. [DETAILS AS NEEDED] Please clarify whether this [conference proceeding or publication] was peer-reviewed and formally published. If this work was previously peer-reviewed and published, in the cover letter please provide the reason that this work does not constitute dual publication and should be included in the current manuscript.

***Portions of these data will be presented at the 2023 Society of Behavioral Medicine Annual Meeting in April. This conference presentation was not peer-reviewed, but the abstract will be published alongside other conference abstracts in an issue of the Annals of Behavioral Medicine. It has not been formally peer-reviewed or published as a journal article elsewhere.*** ***Thus, we do not believe this constitutes dual publication.***

5. We note that you have indicated that data from this study are available upon request. PLOS only allows data to be available upon request if there are legal or ethical restrictions on sharing data publicly. For more information on unacceptable data access restrictions, please see [http://journals.plos.org/plosone/s/data-availability#loc-unacceptable-data-access-restrictions](https://urldefense.com/v3/__http:/journals.plos.org/plosone/s/data-availability*loc-unacceptable-data-access-restrictions__;Iw!!LLK065n_VXAQ!m7EDcJO_qvNMzAW5J2VFwpom5QgS8ZuqJuGMBNrX_oAhJO6UDxSbBmlloCVTbQe-0nfowWBlsTOq6Y90nTo$).

In your revised cover letter, please address the following prompts:

a) If there are ethical or legal restrictions on sharing a de-identified data set, please explain them in detail (e.g., data contain potentially sensitive information, data are owned by a third-party organization, etc.) and who has imposed them (e.g., an ethics committee). Please also provide contact information for a data access committee, ethics committee, or other institutional body to which data requests may be sent.

***As mentioned in our previous submission, data cannot be shared publicly due to ethical restrictions. The first author began the process of redacting the transcripts in preparing this manuscript for initial submission and, after discussing with the IRB staff, realized that the transcripts contain information that would make the participants identifiable. This identifiable information includes their employment history, where they met their spouse, the first names of all their closest family members and the towns where they live, their doctors, etc. Though this information is not traditionally considered identifiable, when viewed in combination, these participants could certainly be identified. Redacting these transcripts would leave them nearly unreadable and therefore not useful to other researchers. We would be happy to provide a transcript that was redacted as an example. A suitable solution seemed to be to allow researchers to request access to anonymized data excerpts as provided by the first author.***

b) If there are no restrictions, please upload the minimal anonymized data set necessary to replicate your study findings as either Supporting Information files or to a stable, public repository and provide us with the relevant URLs, DOIs, or accession numbers. For a list of acceptable repositories, please see [http://journals.plos.org/plosone/s/data-availability#loc-recommended-repositories](https://urldefense.com/v3/__http:/journals.plos.org/plosone/s/data-availability*loc-recommended-repositories__;Iw!!LLK065n_VXAQ!m7EDcJO_qvNMzAW5J2VFwpom5QgS8ZuqJuGMBNrX_oAhJO6UDxSbBmlloCVTbQe-0nfowWBlsTOqfkKOx1Q$).

We will update your Data Availability statement on your behalf to reflect the information you provide.

6. Please review your reference list to ensure that it is complete and correct. If you have cited papers that have been retracted, please include the rationale for doing so in the manuscript text, or remove these references and replace them with relevant current references. Any changes to the reference list should be mentioned in the rebuttal letter that accompanies your revised manuscript. If you need to cite a retracted article, indicate the article’s retracted status in the References list and also include a citation and full reference for the retraction notice.

***We have reviewed the reference list and ensured that it is complete and correct.***

Additional Editor Comments:

We would expect qualitative studies to include the following: 1) defined objectives or research questions; 2) description of the sampling strategy, including rationale for the recruitment method, participant inclusion/exclusion criteria and the number of participants recruited; 3) detailed reporting of the data collection procedures; 4) data analysis procedures described in sufficient detail to enable replication; 5) a discussion of potential sources of bias; and 6) a discussion of limitations.

***We believe we have addressed the components mentioned above with the current revision, including defined objectives and research questions (line 122), description of the sampling strategy (line 148), data collection procedures (line 162), data analysis procedures (line 202), potential sources of bias (line 225), and limitations (line 626).***

[Note: HTML markup is below. Please do not edit.]

Reviewers' comments:

Reviewer #1: As authors themselves have identified that this small sample size may not be representative of group. Use of black and while may be avoided and better use words like race/ Africans origin/ south American origin/ north American origin/ Hispanic/ Negros/ Indiana/ in place of black and white. The analysis / inference may be rewritten based on corrections to avoid language sounding racism . A simple method to assess and report thematic saturation in qualitative research
Greg Guest, plos paper should be read to report - specially point number 26 in table 1 showing how to summaries data-driven saturation studies may yield better presentation. abstract also need refining accordingly. oral testimony should have been grouped by making categories on the basis of responses, however that will require increasing the sample size.

***Thank you for your thoughtful and detailed review. We will address each of your points in turn.***

***As part of our demographic survey, we asked participants to identify themselves and gave them the following options: 1) White; 2) Black or African American; 3) American Indian or Alaska Native; 4) Middle Eastern or North African; 5) Asian, Native Hawaiian or Other Pacific Islander; or 6) Some other race, ethnicity, or origin. All of the participants in our study identified themselves as either White or Black/African American. Given that some Black Americans are of African descent and some are of Caribbean descent, the descriptor “Black” is considered to be broadly inclusive of both sets of racial groups. Neither Black nor White are considered to be derogatory or racist terms in and of themselves.***

***Given that the current study employed a qualitative design, we took special care in the initial manuscript to try to ensure that we did not draw any general conclusions about differences in behavior between Black and White racial groups. Instead, we tried to highlight the differences in behavior between the Black and White participants that we directly observed. However, your comment was a welcome reminder to review the paper and correct any statements that generalized too far beyond the participant sample. For example, on line 621, the previous manuscript read, “If support partners engage in supportive behaviors that threaten an individual’s autonomy but also highlight that they are providing support because they love and care about them, this may lead to beneficial outcomes for Black participants in particular.” We changed this statement to be more exploratory, stating: “Future research should continue to explore whether perceptions of love and care might mitigate potential detrimental effects of support behaviors that appear to threaten autonomy for Black individuals with type 2 diabetes.”***

***Thank you for sharing the paper by Greg Guest, Emily Namey, and Mario Chen (2020). Though not a standard method used in qualitative research for determining thematic saturation, it was extremely informative to learn about a new method that could be used in the future. Interestingly, the paper we used as a benchmark for determining thematic saturation was written by the same authors (Namey et al., 2016). Thus, we believe sample size was determined appropriately in our study and followed guidance set by experts in the field.***

***You also mentioned that “oral testimony should have been grouped by making categories on the basis of responses.” Our analytic process involved creating coding categories based on participants’ responses, which led to the themes we reported in the manuscript. Our process seems to match what you are requesting. However, we welcome clarification so that we can thoroughly address your comments.***

Reviewer #2: Overall, this is a very well written manuscript and timely. Below are two very minor recommendations to enhance the manuscript for the readership:

Line 55, typically diabetes complications are described in addition to comborbidities. Consider rephrasing to diabetes related complications, and comorbid conditions such as cardiovascular disease and chronic kidney disease.

***Thank you for your comment. We have changed the wording to refer to cardiovascular disease and chronic kidney disease as diabetes-related comorbidities, and we referenced diabetes-related complications like retinopathy and amputation separately.***

Please provide rationale for using Kahn and Antonucci’s convoy model. The current description is very helpful however additional information is warranted. Specifically, it will be helpful for readers to know a brief background of this model, what is the purpose of the model, and is it standard use in qualitative methods?

***Point well taken. We have added a sentence to better contextualize our use of the convoy model while trying to keep our comments about it brief, as the convoy model is merely used as a cognitive aid for helping participants recall their relationships. See the additional statement underlined below:***

***“To help participants focus on and recall specific support interactions they remembered having with their close family, the interviewer presented the participant with a visual depiction of Kahn and Antonucci’s (33) convoy model. Traditionally viewed as a heuristic framework for understanding social relationships, some researchers have suggested it may be a useful cognitive aid for discussing relationships within the context of qualitative interviews. (34)”***
