# Supplementary material for: Intrusive social support among Black and White individuals with type 2 diabetes: A “Control issue” or a sign of “Concern and love”?
Source: PLoS One. 2023 Aug 8;18(8):e0288258. doi: 10.1371/journal.pone.0288258 (PMC10409292; doi:10.1371/journal.pone.0288258)
Supplement: Attachment — Submitted filename: Response to Reviewers.docx [file pone.0288258.s005.docx]

PONE-D-23-00304R1

***We thank the editor for their time and effort in providing us with feedback on the current manuscript. Our response to each comment is italicized and bolded.***

Please change the consent to written consent.
***We have done so.***

Also I cannot see whether you have used COREQ checklist. Please can you let me know whether you have submitted this and if not please submit the COREQ checklist.

***We really appreciate this reminder. We have submitted the COREQ checklist and revised the manuscript as needed to comply with the checklist.***
